# Yacon root is a functional food beneficial for human health: a meta-analysis of clinical trials

**DOI:** 10.3389/fnut.2025.1739768

**Published:** 2025-12-11

**Authors:** Ling-Hui Pan, Zhong-Wei Yao, Wei-Feng Hu, Hui-Tian Jia, Wen-Lu Ren, Pan-Pan Wang, Su-ping Ling, He Zhu

**Affiliations:** 1 Taizhou School of Clinical Medicine, Nanjing Medical University, Taizhou, China; 2 Phase I Clinical Research Center, The Affiliated Taizhou People's Hospital of Nanjing Medical University, Taizhou, China; 3 Department of Pharmaceutical Analysis, Third Clinical Medical College, Nanjing University of Chinese Medicine, Nanjing, China; 4 Department of Metabolomics, Affiliated Hospital of Integrated Traditional Chinese and Western Medicine, Nanjing University of Chinese Medicine, Nanjing, China

**Keywords:** yacon root (YR), multi-function, meta-analysis, functional food, metabolic regulation

## Abstract

Yacon (*Smallanthus sonchifolius*) is a perennial herbaceous plant in the Asteraceae family, native to the Andean region of South America. Yacon root (YR) is considered to be a functional food but with inconsistent conclusions. We hypothesized that YR beneficially modulates human health. This study aims to evaluate the effects of YR on human health through clinical trials to determine whether YR can be classified as a functional food. We systematically searched PubMed, Cochrane Library, Springer, and Web of Science for clinical outcomes related to YR from 2000 to 2025. The health benefits of YR were evaluated through a meta-analysis. Twelve studies were included in the analysis. The pooled results showed that YR significantly reduced BMI [SMD = −0.81, 95%CI (−1.54, −0.08)], decreased stool pH [SMD = −1.12, 95%CI (−1.61, −0.62)], softened stools [SMD = 0.94, 95%CI (0.08, 1.80)], facilitated defecation [SMD = 4.03, 95% CI (0.54, 7.52)], improved fiber consumption patterns [SMD = 0.58, 95%CI (0.18, −0.99)]. Additionally, YR tends to reduce body weight, waist circumference, postprandial blood glucose and triglyceride levels, and dietary intake of energy, carbohydrates, fats, and proteins. Collectively, YR shows evidence supporting its classification as a functional food for weight control, constipation relief. These effects may be ascribed to active components, such as inulin-type fructans and phenolic compounds. However, the limited quality of the included studies indicates that future high-quality clinical trials are necessary to confirm YR’s effectiveness.

**Systematic review registration:**

https://www.crd.york.ac.uk/prospero/display_record.php?ID=CRD42024606929, identifier (CRD42024606929).

## Graphical abstract

**Figure:**
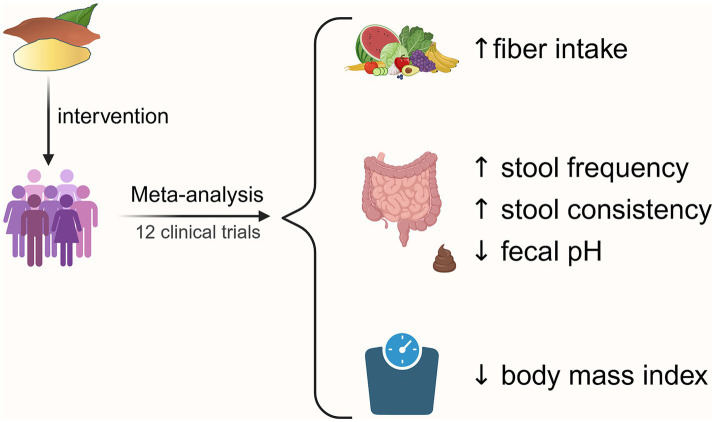
YR is considered a functional food, but its clinical effects remain inconsistent. This meta-analysis of 12 randomized clinical trials showed that YR intake significantly reduced BMI and fecal pH, and increased stool frequency, stool consistency, and fiber intake. These findings support the classification of YR as a functional food for weight control and constipation relief. YR, Yacon root; BMI, Body mass index.

## 1 Introduction

Yacon (*Smallanthus sonchifolius*) is a perennial herb in the Asteraceae family that native to the Andes Mountains of South America from Venezuela to northwestern Argentina and currently spread all over the world, such as New Zealand, Japan, China, and Germany (1). Yacon is well-known for its edible tuberous root, which has a sweet taste with crunchy texture resembling to apple (2). Yacon root (YR) can be consumed as raw fruit and also be processed into air-dried tuber slices, juice, flour, syrup and YR containing food (3). In fact, YR and its processed products have been become worldwide favorite foods (4).

Preclinical experiments have shown that YR has positive effects on weight control (5), lipid metabolism (6), blood glucose regulation (6, 7), intestinal health (8), bone health (9, 10) and oxidative stress (11), and therefore been considered to be a potential functional food. However, further clinical trials yielded different results and even contradictory conclusion. For example, some clinical studies indicated that YR can reduce blood glucose and insulin levels (12, 13) and regulate blood lipids (14, 15), but other clinical experiments support opposite opinions (12, 14, 16). Therefore, it is necessary to verify whether YR could be used as a functional food.

Meta-analysis is a powerful tool to analyze the inconsistent results from different trials and further obtain more scientific conclusion (17). In this study, a meta-analysis was performed to investigate the effects of YR on human health to find the actual function of the YR and further access whether YR is a functional food. We hypothesized that YR beneficially modulates human health, particularly in aspects related to weight control and bowel function.

## 2 Methods

### 2.1 Study registration

The meta-analysis was conducted in accordance with the Preferred Reporting Items for Systematic Reviews and Meta-Analyses (PRISMA) guidelines (18) and registered on PROSPERO (Registration Number: CRD42024606929).

### 2.2 Search strategy

A comprehensive electronic database search strategy was constructed to identify the effects of the YR on human health. The systematic search was performed on PubMed, Cochrane Library, Springer link, and Web of Science using “yacon” (MeSH) or “Smallanthus sonchifolius” (MeSH) terms from 2000 to November 2025, and major Chinese databases, including CNKI, Wanfang, and VIP, using “xuelianguo,” “yagong,” “XLG,” “yacon,” and “Smallanthus sonchifolius” terms from inception to November 2025. No search filters or limits were applied.

### 2.3 Literature selection

The PICOS (population, intervention, control, and outcomes) model, which outlines the inclusion and exclusion criteria, was used to select the eligible studies for the meta-analysis (Table 1).

**Table 1:** Eligibility criteria for study inclusion in the meta-analysis of YR clinical trials based on the PICOS framework.

Parameter	Description
Population (P)	Healthy or metabolically disturbed adults
Intervention (I)	YR or YR processed products
Comparator(C)	Placebo
Outcomes (O)	Weight, waist circumference, BMI, blood glucose indices, blood lipid indices, bowel movement status, and Nutrient intake
Study design (S)	Placebo-controlled clinical trials

Inclusion criteria were as follows: (1) availability of full-text articles; (2) clinical trials; (3) healthy people or people with metabolic disorders aged over 18 years; (4) single YR either in pure or standardized extract forms as a standalone intervention (5) reporting the mean and standard deviation (or standard error) or providing necessary data for these values at baseline and postintervention in both intervention and control groups; (6) reporting the sample size.

Exclusion criteria were as follows: (1) had participants involved adolescents; (2) involved the combined effects of other interventions; (3) presented insufficient data on the primary outcomes in both intervention and control groups; (5) reported in languages other than English; (6) showed only sensory outcome measures and (7) repeated publications of the same population in which the data were included for once unless there were different outcome measures.

### 2.4 Data extraction

Two investigators independently extracted data from the included studies after reviewing the titles, abstracts, and full texts. Data were extracted from the graphs using GetData Graph Digitizer 2.25 (19). A third investigator independently assessed and verified all data extraction. The researchers discussed any inconsistencies and disagreements, and a consensus was reached regarding the opinion of the researcher (Dr. Zhu), if necessary.

General information (authors, journal, publication year, and study design), participant information (age, gender ratio, sample size, participant descriptions, and baseline characteristics), intervention and control details (type, dosage of YR, and study duration), and outcome measures (weight, waist circumference, BMI, blood glucose indices, blood lipid indices, bowel movement status, and Nutrient intake) were extracted from the eligible literatures.

### 2.5 Methodological quality evaluation

The Cochrane Handbook was utilized for the evaluation of risk bias. Two investigators evaluated the methodological quality from selection bias, performance bias, detection bias, attrition bias, reporting bias, and other biases. The methodological assessment for each trial was categorized using nominal scales: “yes” indicated a low risk of bias, “unclear” indicated an uncertain risk, and “no” indicated a high risk of bias.

### 2.6 Statistical analysis

Forest plots were generated only when two or more studies were available, in accordance with the Cochrane Handbook. If only one study was available, the findings were summarized narratively.

All the meta-analyses were performed using Stata software (version 17.0, Stata Corporation, College Station, TX) and Review Manager (RevMan) 5.4 software (Cochrane Collaboration, Oxford, UK). Mean difference (MD) was calculated with 95% confidence interval (CI), and *I*^2^ test was used to evaluate the heterogeneity of the data. Considering the significant heterogeneity between different studies, a random-effects meta-analysis was performed to analyze the data by pooling the outcomes of the included studies. Subgroup analysis was conducted to investigate the effects of various variables on clinical outcomes.

## 3 Results

### 3.1 Research screening

**Figure 1:**
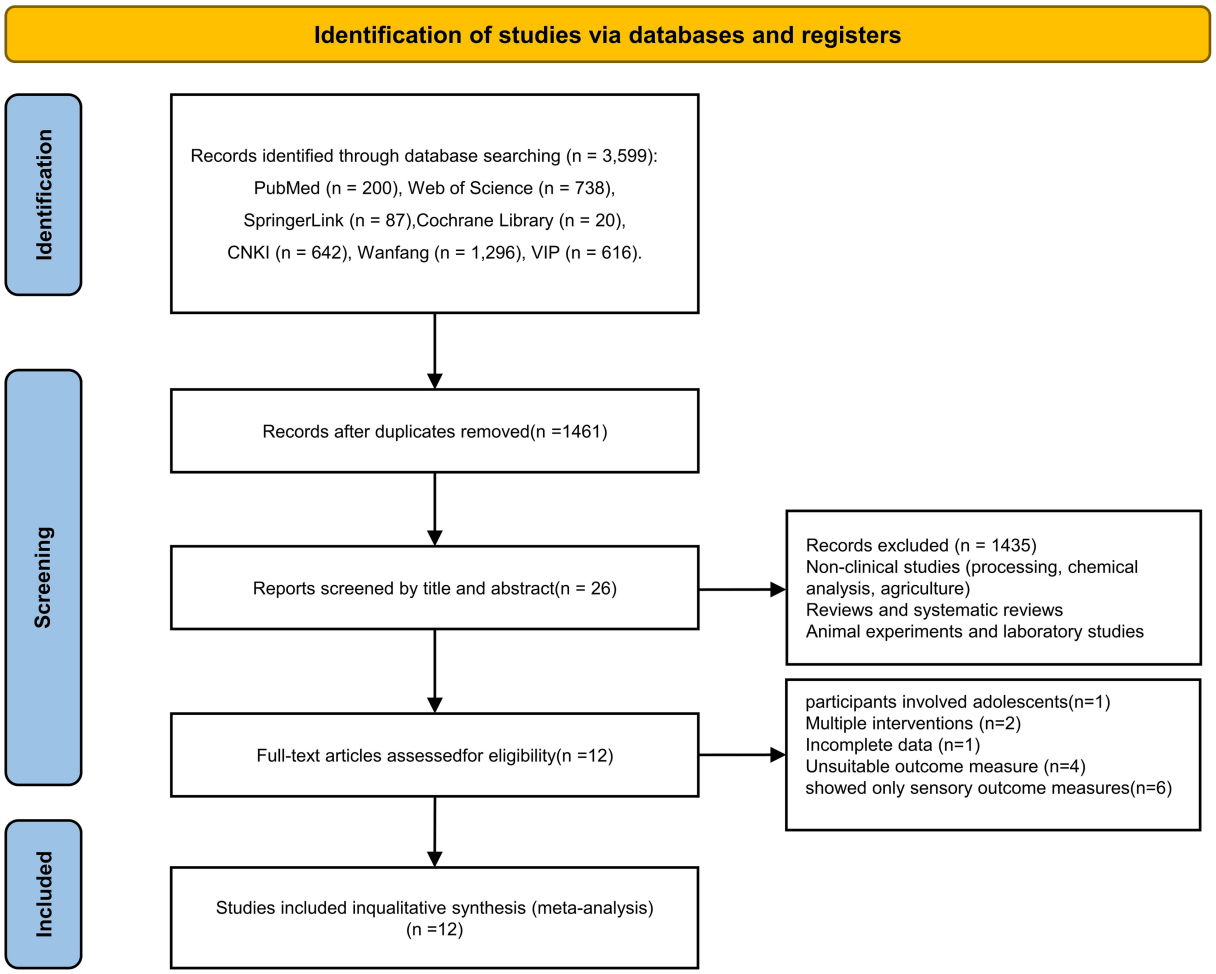
Flow diagram of study selection process. A total of 12 clinical trials were finally included in the meta-analysis.

The screening process for eligible studies, as illustrated in the PRISMA flowchart, is presented in Figure 1. A total of 3,599 articles were identified through searching databases, resulting in 1,461 records after excluding 2,138 duplicates. Subsequently, 1,449 articles were excluded due to non-compliance with the inclusion criteria, and ultimately 12 eligible articles were selected for eligibility.

### 3.2 Research characteristics

The characteristics of the included studies are summarized in Table 2. Among the included 12 studies (12–16, 20–26), four employed a self-controlled design (*n* = 123), and eight used placebo-controlled design (*n* = 302). Each study included between 15 and 55 subjects. The ages of participants ranged from 24.87 ± 2.75 to 67.11 ± 6.12 years. The studies originated from various geographical locations, including Asia: Japan (*n* = 1), South America: Peru (*n* = 1), Argentina (*n* = 1), and Brazil (*n* = 9). The studied population consisted of healthy subjects, excess-weight adult volunteers, patients with type 2 diabetes, and elderly subjects. The intervention duration varied from 1 day to 5 months. The dosage was calculated based on the FOS in YR, ranging from 6.4 g to 14 g FOS/day. Participants received interventions through yacon syrup, freeze-dried powdered yacon (FDY), a yacon-based product (YBP), yacon flour, and yacon shake. The primary outcome measures included BMI, body weight, waist circumference, stool frequency, stool consistency, fecal pH and short-chain fatty acids, as well as glucose, insulin and HOMA-IR levels, lipid and lipoprotein levels and nutrient intake. 3.3 Risk of bias of the included trials.

**Table 2:** Characteristics of clinical trials included to evaluate the effects of YR on human health.

Study	Country	Population	Age (Mean ± SD)		Gender		Duration (*d*)	Intervention	Dosage (g FOS/d)	Outcome measure
Geyer et al. 2008 (26)	Peru	Health	29.3 ± 4.9		8 M/8 F		14	Yacon syrup	6.4	a
Genta et al. 2009 (15)	Argentina	Obesity and dyslipidemia perimenopause	41 ± 7 (YG)	40 ± 9 (PG)	20 F (YG)	15 F (PG)	120	Yacon syrup	10	b, c, d
Satoh et al. 2014 (25)	Japan	Patients with type 2 diabetes	66.9 ± 1.3 (YG)	65.6 ± 1.6 (PG)	29 (YG)	27 (PG)	150	Yacon	8	b, c, d
Scheid et al. 2014 (13)	Brazil	Elders	67.11 ± 6.12 (YG)	67.11 ± 5.53 (PG)	37 (YG)	35 (PG)	63	FDY	7.4	c, d
de Souza Lima Sant'Anna et al. 2015 (24)	Brazil	Constipated individuals	39.83 ± 18.51 (YG)	39.71 ± 17.62 (PG)	6 M/42 F		30	YBP	10	a, e
Gomes da Silva et al. 2017 (23)	Brazil	Health	27.6 ± 5.1		10 F/10 M		14/1	Yacon syrup	7.4	f
Rocha et al. 2018 (16)	Brazil	Health	24.87 ± 2.75		1 M/14 F		1	Yacon	7.4	c
Machado et al. 2019(14)	Brazil	Obesity	29.77 ± 7.26 (YG)	32.92 ± 9.68 (PG)	5 M/8 F (YG)	6 M/7 F (PG)	42	Yacon flour	8.7	f
Adriano et al. 2019 (12)	Brazil	Health and obesity	Normal: 24.5 ± 4.96; Obese: 27.0 ± 4.89		40F		1	Yacon syrup	14	c, d
Dionísio et al. 2020 (20)	Brazil	Health	41.7 ± 8.9 (YG)	40.1 ± 8.0 (PG)	10 F/5 M (YG)	11 F/4 M (PG)	14	Yacon syrup	8.7	b, c, d
Machado et al. 2021 (22)	Brazil	Obesity	29.77 ± 7.26 (YG)	32.92 ± 9.68 (PG)	5 M/8 F (YG)	11 F/4 M (PG)	42	Yacon flour	8.7	e
Ribeiro et al. 2021 (21)	Brazil	Obesity	29.77 ± 7.26 (YG)	32.92 ± 9.68 (PG)	5 M/8 F (YG)	6 M/7 F (PG)	42	Yacon flour	8.7	b, c, d

a: defecation status; b: anthropometric evaluation; c: clinical parameters related to glycemic; d: clinical parameters related to lipid; e: fecal pH and short chain fatty acid (SCFAs); f: nutrient intake. M, male; F, female. FDY: freeze-dried powdered yacon; YBP: yacon-based product. YG: Yacon group; PG: placebo group.

**Figure 2:**
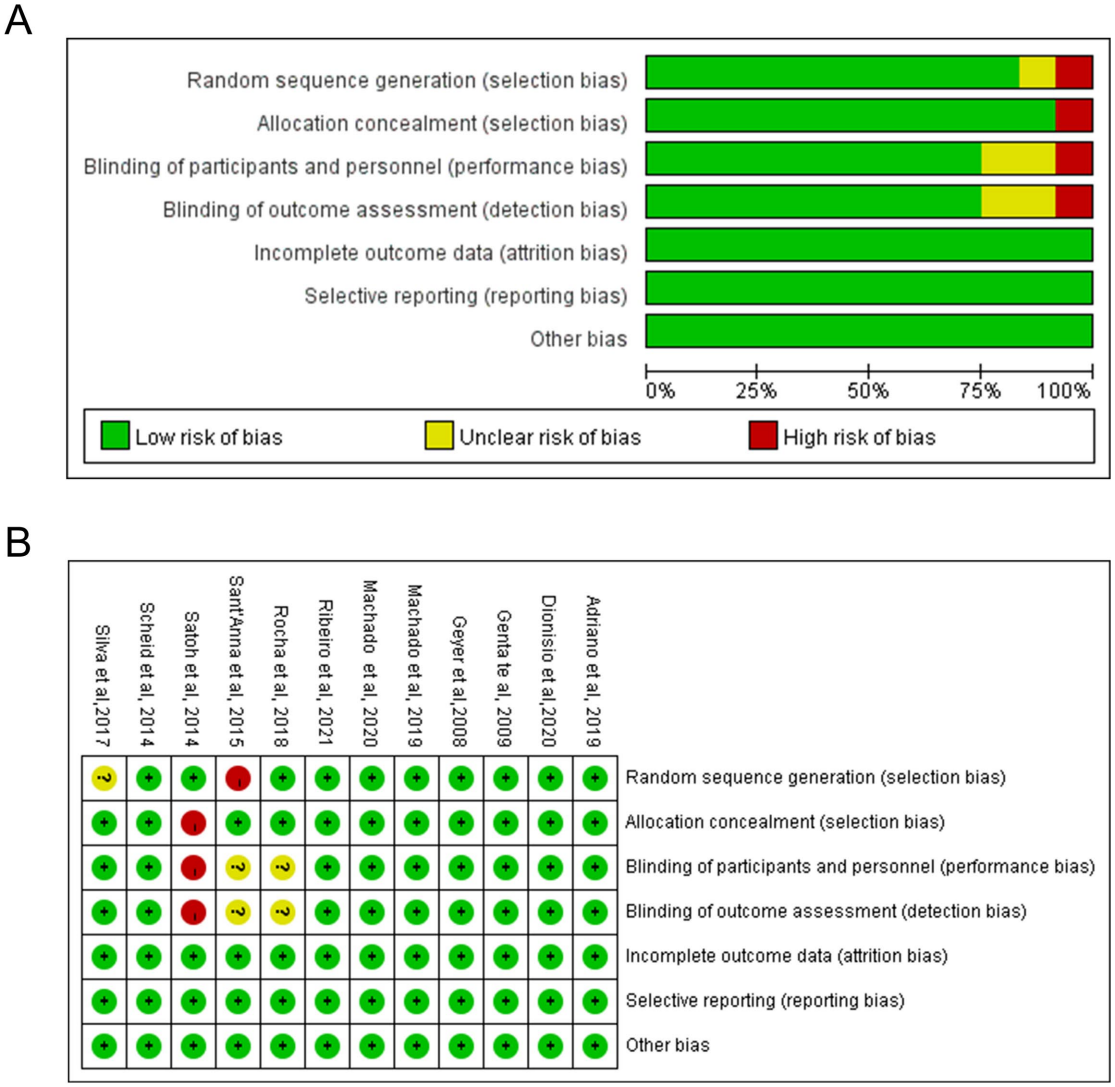
Risk of bias assessment of the clinical trials included in the meta-analysis of YR supplementation. **(A)** Risk of bias graph showing the percentage of studies rated as low risk, some concerns, or high risk across different domains. **(B)** Risk of bias summary for each included study across all domains. Green indicates low risk of bias, yellow indicates some concerns, and red indicates high risk of bias.

The risk of bias of the 12 studies was assessed using the Cochrane Collaboration tool. In selection bias, 10 trials employed random allocation schemes, classifying low risk. Additionally, 10 trials reported adequate allocation concealment, also deemed low risk. Nine studies implemented double-blind protocols, which were considered low risk for both performance and detection biases. All studies presented complete data, leading to a low risk assessment for attrition bias. In summary, out of the 12 studies, 9 trials exhibited an overall low risk of bias, with no areas of high or unclear risk identified (Figure 2).

### 3.3 Anti-obesity effect

#### 3.3.1 BMI

Five studies utilized BMI as an outcome measure. BMI changes before and after the intervention were extracted for meta-analysis, revealing a significant decrease in the experimental group (SMD = −0.81, 95%CI (−1.54, −0.08), *p* = 0.03, *I*^2^ = 82.6%) (Table 3; Supplementary Figure S1A). Subgroup analysis based on subject type revealed that YR significantly reduced BMI in overweight and type 2 diabetes participants, respectively (Supplementary Figure S1B).

**Table 3:** Results of the meta-analysis of YR supplementation on anthropometric, metabolic, and bowel function outcomes in humans.

Outcomes	Experimental (*n*)	Control (*n*)	SM	SMD	[95% CI]		*p*	*I^2^* (%)
BMI*	101	94	REM	−0.81	−1.54	−0.08	0.03	82.6
Body weight*	101	94	REM	−0.56	−1.18	0.06	0.08	76.5
Waist circumference	48	43	REM	−1.07	−2.47	0.32	0.13	89.0
Stool frequency	60	55	REM	4.03	0.54	7.52	0.02	97.0
Stool consistency	53	53	REM	0.94	0.08	1.80	0.03	76.5
fecal pH	37	37	REM	−1.12	−1.61	−0.62	0.00	0.0
Acetate*	37	37	REM	−0.04	−0.49	0.42	0.88	0.0
Propionate*	37	37	REM	−0.08	−0.53	0.38	0.74	0.0
Butyrate*	37	37	REM	1.07	−1.12	3.25	0.34	94.2
Fasting glucose*	114	105	REM	0.45	−0.57	1.47	0.39	91.8
Fasting insulin	85	78	REM	−0.55	−1.81	0.72	0.40	92.1
HOMA-IR*	99	90	REM	−0.69	−2.21	0.82	0.37	95.0
Postprandial glucose	55	55	REM	0.32	−0.69	0.06	0.10	0.0
Fasting total Cholesterol	85	78	REM	0.02	−0.29	0.33	0.90	0.0
Fasting LDL-c*	114	105	REM	0.12	−0.76	1.01	0.79	89.5
Fasting HDL-c*	114	105	REM	−0.04	−0.31	0.22	0.76	0.0
Fasting triglyceride*	114	105	REM	0.54	−0.39	1.46	0.25	90.1
Postprandial triglyceride	40	40	REM	−0.13	−0.57	0.31	0.55	0.0
Fiber*	50	48	REM	0.58	0.18	0.99	0.01	0.0
Energy	50	48	REM	−0.16	−0.56	0.24	0.43	0.0
Carbohydrates*	65	63	REM	−0.08	−0.42	0.27	0.67	0.0
Fat	65	63	REM	−0.15	−0.50	0.20	0.39	0.0
Protein*	65	63	REM	−0.12	−0.47	0.23	0.49	0.0

SMD, standardized mean difference; CI, confidence intervals; SM, statistical method; REM, random effects model; FEM, fixed effects model. *Data was extracted using GetData Graph Digitizer.

#### 3.3.2 Body weight

Four studies measured weight changes before and after the intervention. The meta-analysis indicated a trend toward weight loss, but no significant difference in weight change between the experimental and control groups (SMD = −0.56, 95%CI (−1.18, 0.06), *p* = 0.08, *I*^2^ = 76.5%) (Table 3; Supplementary Figure S2A). Further subgroup analysis by subject population indicated that YR tends to reduce body weight in both healthy and overweight participants (Supplementary Figure S2B).

#### 3.3.3 Waist circumference

Three studies involving overweight adult volunteers were used to examine the effect of YR on waist circumference. Compared to the control group, YR consumption showed a tendency to reduce waist circumference in overweight individuals, although without significant differences (SMD = −1.07, 95%CI (−2.47, 0.32), *p* = 0.13, *I*^2^ = 89.0%) (Table 3; Supplementary Figure S3A). Subgroup analysis by subject population revealed that YR has a more significant impact on waist circumference in overweight participants (Supplementary Figure S3B).

### 3.4 Stool frequency, stool consistency, fecal pH and short−chain fatty acids

Three studies were pooled to assess the effect of YR on the stool frequency in subjects. Compared with the control group, the stool frequency in the YR group was significantly increased (SMD = 4.03, 95% CI (0.54, 7.52), *p* = 0.02, *I*^2^ = 97.0%) (Table 3; Supplementary Figure S4A).

Three studies assessed stool consistency using the Bristol Stool Form Scale, and the meta-analysis results showed that YR consumption significantly softened the stool consistency of participants (SMD = 0.94, 95%CI (0.08, 1.80), *p* = 0.032, *I*^2^ = 76.5%) (Table 3; Supplementary Figure S4B).

Two studies evaluated the effect of YR on fecal pH. The meta-analysis revealed a significant decrease in fecal pH in the YR interventional group (SMD = −1.12, 95%CI = −1.61, −0.62, *p* = 0.000) with no heterogeneity (*I*^2^ = 0.0%) (Table 3; Supplementary Figure S4C).

Two studies analyzed the effect of YR on the contents of short-chain fatty acids (SCFAs) including acetate, propionate, and butyrate. The meta-analysis revealed that no significant differences in SCFAs levels before and after YR intervention but with tendencies: acetate: SMD = −0.04, 95%CI (−0.49, 0.42), *p* = 0.88, *I*^2^ = 0.0%; propionate: SMD = −0.08, 95% CI (−0.53, 0.38), *p* = 0.74, *I*^2^ = 0.0%; butyrate: SMD = 1.07, 95%CI (−1.12, 3.25), *p* = 0.34, *I*^2^ = 94.2% (Table 3; Supplementary Figure S5).

### 3.5 Blood glucose, insulin levels and HOMA-IR

Five studies concerned the changes in fasting blood glucose following YR consumption. Mean values and standard deviations of changes before and after intervention were extracted for meta-analysis. Results indicated YR showed no effects on fasting blood glucose (SMD = −0.55 95%CI (−1.81, 0.72), *p* = 0.4) with high heterogeneity (*I*^2^ = 92.1%) (Table 3; Supplementary Figure S6A). Four studies reported fasting insulin statistics and HOMA-IR, respectively. The meta-analysis revealed no statistical significance in fasting insulin (SMD = −0.55, 95%CI (−1.81, 0.72), p = 0.4, *I*^2^ = 92.1%) and HOMA-IR (SMD = −0.69, 95%CI (−2.21, 0.82), *p* = 0.371, *I*^2^ = 95.0%) between pre- and post-intervention (Table 3; Supplementary Figures S6B,C).

Two studies (12, 16) containing three trials investigated the effect of YR on postprandial blood glucose. Meta-analysis results indicated no significant difference between experimental and control groups (SMD = −0.32, 95%CI (*−*0.69, 0.06), *p* = 0.10, *I*^2^ = 0.0%) but with decrease tendency (Table 3; Supplementary Figure S7A).

### 3.6 Levels of blood lipids and lipoproteins

Three studies assessed fasting total cholesterol, and five studies examined fasting HDL-c, LDL-c and triglycerides before and after intervention. The meta-analysis showed that YR did not reduce fasting total cholesterol, HDL-c, LDL-c and triglycerides but with regulatory tendencies (total cholesterol: SMD = 0.02, 95% CI (−0.29, 0.33), *p* = 0.90, *I*^2^ = 0.0%; LDL-c: SMD = 0.12, 95%CI (−0.76, 1.01), *p* = 0.76, *I*^2^ = 89.5%; HDL-c: SMD = −0.04, 95%CI (−0.31, 0.22), *p* = 0.76, *I*^2^ = 0.0%; triglycerides: SMD = 0.54, 95% CI (−0.39, 1.46), *p* = 0.25, *I*^2^ = 90.1%) (Table 3; Supplementary Figure S8).

One study with two trials evaluated the effect of YR on postprandial triglyceride levels in both healthy and overweight individuals. The meta-analysis showed that YR decreased postprandial triglyceride levels in a certain degree (SMD = −0.13, 95%CI (*−*0.57, 0.31), *p* = 0.55, *I*^2^ = 0.0%) (Table 3; Supplementary Figure S7B).

### 3.7 Nutrient intake

Two studies assessed fiber intake before and after intervention. The meta-analysis revealed fiber intake was significantly increased in interventional group (SMD = 0.58, 95%CI (0.18, 0.99), *p* = 0.01, *I*^2^ = 0.0%). Two studies analyzed energy intake before and after intervention. The meta-analysis indicated energy intake was not significantly changed, but with a decrease tendency (SMD = −0.16, 95%CI (*−*0.56, 0.24), *p* = 0.42, *I*^2^ = 0.0%). Three studies reported carbohydrate, fat, and protein intake before and after intervention. The meta-analysis showed the intake of carbohydrate, fat and protein in interventional group showed a decreasing trend (Carbohydrates: SMD = −0.08, 95%CI (−0.42, 0.27), *p* = 0.67, *I*^2^ = 0.0%; Fat: SMD = −0.15, 95%CI (−0.50, 0.20), *p* = 0.39, *I*^2^ = 0.0%; Protein: SMD = −0.12, 95%CI (−0.47, 0.23), *p* = 0.49, *I*^2^ = 0.0%) (Table 3; Supplementary Figure S9).

## 4 Discussion

This meta-analysis showed that YR significantly reduced the BMI and fecal pH, and increased the stool frequency, stool consistency and fiber intake, indicating that YR is a kind of functional food. These findings confirmed our hypothesis that YR beneficially modulates human health, particularly in aspects related to weight control and bowel function. Generally, the functional effects of foods are determined by their active ingredients. As shown in Table 4, YR is rich in inulin-type fructans, polyphenolic compounds and other ingredients, which might contribute to its functional roles *in vivo*.

**Table 4:** Identified chemical constituents and biological activities of YR.

Compound	Chemical formula	Biological effects	References
Caffeic acid	C_9_H_8_O_4_	Anti-inflammatory, antioxidant, Immunomodulatory, anti-hyperglycemic, Antidepressant, anticancer, antiviral	(44–46)
Chlorogenic acid	C_16_H_18_O_9_	Anti-inflammatory, antioxidant, antidiabetic	(47, 48)
Ferulic acid	C_10_H_10_O_4_	Anti-inflammatory, anti-hyperglycemic, antimicrobial, anticancer, anti-apoptotic, anti-platelet	(49–51)
3,5-Dicaffeoylquinic acid	C_25_H_22_O_12_	Antioxidant	(52)
2,3,5- or 2,4,5-tricaffeoylaltraric acid	C_33_H_30_O_16_	Antioxidant, α-glucosidase inhibitory, anti-hyperglycemic	(52)
Quercetin	C_15_H_10_O_7_	Antioxidant, anti-inflammatory, anti-proliferative, anti-hyperglycemic, anticancer, antiviral	(40, 53)
Citric acids	C_6_H_8_O_7_	Catalyzes partial hydrolysis of inulin to FOS, Antibacterial	(54, 55)
Malic acids	C_4_H_6_O_5_	Antibacterial	(55)
Fumaric acids	C_4_H_4_O_4_	Antioxidant	(56)
Quinic acid	C_7_H_12_O_8_	α-glucosidase inhibitor, reduces blood lipids	(57, 58)
p-Coumaric acid	C_9_H_8_O_4_	Anti-inflammatory, antioxidant, cardioprotective, neuroprotective	(59)
Kaempferol	C_15_H_14_O_6_	Anti-inflammatory, anticancer, antibacterial, antifungal, antiprotozoal, alleviates obesity	(60, 61)
Isorhamnetin	C_16_H_12_O_7_	cardiovascular protection, anti-tumor, anti-Inflammatory, antioxidant, organ protection, obesity prevention	(62)
Fructooligosaccharides (FOS)	(C_6_H_10_O_5_) _n-2_	Prebiotic effects, alleviates obesity, antioxidant, anti-cancer, anti-inflammatory, anti-hyperglycemic, anti-bacterial, protect the intestine	(63–67)
1-kestose (GF2)	C_12_H_22_O_11_		
Nystose (GF3)	C_18_H_32_O_16_		
1F-β-fructofuranosyl nystose (GF4)	C_24_H_40_O_22_		
Inulin	(C_6_H_10_O_5_) _n_	Prebiotic effect, regulates lipid metabolism, laxative, alleviates obesity, anti-hyperglycemic, anti-inflammatory, anti-cancer, promotes mineral absorption	(29)
Fructose	C_6_H_12_O_6_	Provides energy, regulates blood glucose	
Glucose	C_6_H_12_O_6_	Provides energy, regulates blood glucose	
Sucrose	C_12_H_22_O_11_	Provides energy, regulates blood glucose	
L-tryptophan	C_11_H_12_N_2_O_2_	Precursor of serotonin and niacin synthesis, immunomodulatory, anti-inflammatory, antioxidant	
L-Arginine	C_6_H_14_N4O_2_	Improves cardiovascular health, prevents kidney failure	(68, 69)
L-glutamic acid	C_5_H_9_NO_4_	Regulates acid–base balance, treats encephalopathy	(70, 71)
L-proline	C_5_H_9_NO_2_	Prevents endoplasmic reticulum stress, combats *Klebsiella pneumoniae* infections	(72, 73)
L-aspartic acid	C_4_H_7_NO_4_	antitumor	
Asparagine	C_4_H_8_N_2_O_3_	Protein synthesis, neurotransmitter synthesis, anti-inflammatory, antioxidant	
Glutamine	C_5_H_10_N_2_O_3_	Anti-fatigue, immune regulation, metabolism	(74, 75)
Alanine	C_3_H_7_NO_2_	Protein synthesis, energy metabolism	
Threonine	C_4_H_9_NO_3_	Protects intestinal mucosa, protein synthesis	(76, 77)
Valine	C_5_H_11_NO_2_	Anti-inflammatory, intestinal protection	(78)
Potassium	K	Maintains fluid balance, regulates osmotic pressure	(79, 80)
Phosphorus	P	β-lactamase inhibitor, antibacterial	(81, 82)
Calcium	Ca	Regulates calcium signaling, protects bones	(83)
Magnesium	Mg	Assists enzymatic reactions, regulates muscle contraction, blood pressure, insulin metabolism, cardiovascular function, neurotransmission	(84)
Sulfur	S	Anti-inflammatory, antioxidant, prevents chronic diseases	(85)
Sodium	Na	Regulates Na-K ATPase activity	(86)

### 4.1 Inulin-type fructans

Inulin-type fructans, including fructooligosaccharides (FOS, DP < 10) and inulin (DP 2–60), are low-calorie dietary fibers. They can replace high-calorie carbohydrates, reduce energy intake, slow gastric emptying, suppress hunger, and decrease the small intestine’s absorption of sugar and fat. In addition, inulin-type fructans resistant to digestive enzymes could reach colon, where optimize the intestinal microenvironment *via* promoting the generation of beneficial bacteria and production of postbiotics, and reducing the generation of pathogenic bacteria and production of intestinal endotoxin (27, 28). The intestinal microenvironment optimization improves dysbiosis-related conditions, including but not limited to obesity, constipation and metabolic syndrome. For example, beneficial bacteria enhance intestinal barrier function, decrease permeability, and limit the entry of pro-inflammatory substances like lipopolysaccharide (LPS) into the bloodstream, reducing chronic low-grade inflammation associated with obesity (29–31). Meanwhile, SCFAs (mainly acetic, propionic, and butyric acids), one of a type of postbiotics yielded by FOS, regulate various physiological processes in the human body. SCFAs lower colonic pH (32, 33), maintain intestinal moisture and osmotic pressure, increase stool viscosity and moisture, promote defecation, and alleviate constipation (34). They also reduce appetite and food intake by modulating appetite-related hormones, increasing peptide YY levels, and inhibiting growth hormone-releasing peptide and leptin secretion (35–37).

### 4.2 Phenolic compounds

YR contains abundant phenolic compounds, including chlorogenic acid, caffeic acid, ferulic acid, quinic acid, and quercetin, which are bioactive substances crucial for managing chronic conditions like obesity, diabetes, and metabolic disorders. Phenolic compounds mainly exert functional effects through the following manners. (1) Phenolic compounds regulate lipid metabolism. For example, chlorogenic acid inhibits porcine pancreatic lipase (EC 3.1.1.3), reduces lipid absorption, and upregulates peroxisome proliferator-activated receptor (PPAR) mRNA, promoting fat metabolism and reducing weight gain and visceral fat accumulation in the liver (38). Kaempferol inhibits lipogenesis by suppressing Akt and mTORC1 activation, blocking downstream SREBP1C signaling, and directly activating AMPK to further inhibit SREBP1C-mediated adipogenesis (39). Quercetin enhances lipid metabolism and promotes lipolysis through SIRT1 and Akt pathway activation (40). (2) Phenolic substances regulate blood glucose metabolism. For instance, chlorogenic and caffeic acids inhibit *α*-amylase, lowering blood glucose and increasing insulin levels, thereby improving pancreatic *β*-cell function (41). Quercetin improves insulin signaling and lowers blood glucose by promoting Akt and glycogen synthase kinase-3 (GSK-3) phosphorylation while its antioxidant properties reduce oxidative stress and repair damaged pancreatic β-cells (40). Ferulic acid neutralizes streptozotocin toxicity via antioxidant activity, protecting β-cells, promoting their proliferation and insulin secretion, and enhancing hepatic glucose utilization (42). Quinic acid stimulates insulin secretion and regulates blood glucose by mobilizing intracellular calcium ions and increasing the NADPH/NADP^+^ ratio (43).

**Figure 3:**
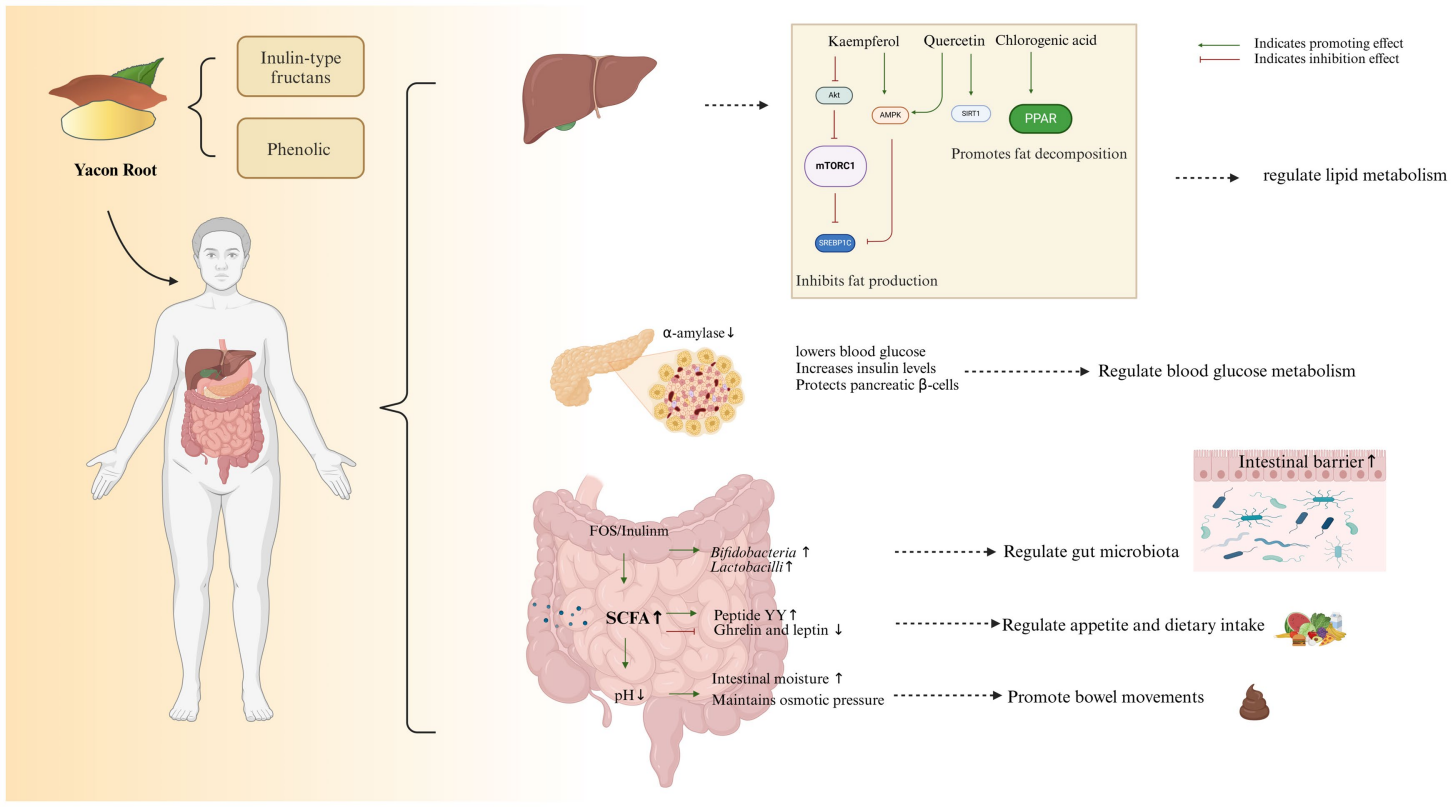
YR, with the active components of inulin-type fructans and phenolic compounds, operates through specific modes of action.

In summary, we speculate that the function of YR may be the result of the combined action of inulin-type oligosaccharides, phenolic substances, etc. in YR (Figure 3). However, the precise mechanisms underlying YR’s clinical effects require further investigation.

## 5 Recommendations

### 5.1 Strength

This study has several strengths. First, we conducted a systematic and comprehensive search across multiple major English-language databases and applied strict, explicit and reproducible inclusion and exclusion criteria. This approach improved the completeness of the literature sources and enhanced the reliability of the findings. Second, only clinical trials using YR as the sole intervention were included, which minimized confounding from other dietary components or combined supplements and allowed a more clinically targeted and accurate attribution of effects. Third, this study integrated multiple outcome dimensions, including anthropometric indicators, bowel function, glucose and lipid metabolism, and dietary intake, providing a systematic evaluation of the potential clinical benefits of YR in weight management, metabolic health, and gut function. Previous studies have suggested that YR may be valuable for individuals with obesity, metabolic disorders, and functional constipation, possibly through its prebiotic effects, appetite regulation, and gut microbiota–mediated metabolic improvements.

### 5.2 Limitations

This study has several limitations. First, for only English-language publications were primarily included, there is a possibility of language bias and retrieval bias due to limited database coverage. We conducted additional searches in CNKI, Wanfang, and VIP using the keywords “xuelianguo,” “yagongguo,” and “XLG,” but the relevant Chinese literature were animal studies, and no randomized controlled clinical trials meeting the inclusion criteria were identified. In addition, nine of the included studies were conducted in Brazil, which may introduce regional bias and affect the generalizability of the findings. Second, the included studies generally had small sample sizes, and the participants varied across healthy subjects, overweight or obese individuals, patients with type 2 diabetes, and elderly populations. Baseline differences among these groups may lead to variations in effect size. Although subgroup analyses suggested that YR may have more pronounced effects in overweight individuals, this observation may be influenced by confounding factors and requires further validation. Finally, some outcomes showed high statistical heterogeneity, indicating substantial differences across studies in terms of intervention form, dosage, duration, and lifestyle background. These factors may affect the stability of the effect estimates, and therefore the results should be interpreted with caution.

### 5.3 Future research directions

The findings of this study indicate that YR, as a safe and natural source of dietary fiber or prebiotics, may be applied in nutritional interventions for individuals with mild obesity, metabolic disturbances, and functional constipation. It may also serve as a dietary option for populations with inadequate fiber intake. In addition, these results provide scientific support for the development of YR-based functional foods. The potential value of YR in personalized and precision nutrition strategies also warrants further exploration.

Given the limited scale and high heterogeneity of the current evidence, future studies should include large-sample, multicenter, and long-term randomized controlled trials, with a particular focus on populations at metabolic risk. These studies should systematically evaluate the dose–response relationship of different formulations (e.g., syrup, powder), dosages, and intervention durations. Furthermore, mechanistic indicators such as gut microbiota and metabolites should be incorporated to identify the most suitable target populations and to elucidate the underlying mechanisms of action.

## 6 Conclusion

YR has been shown to reduce BMI, improve stool pH, consistency, and defecation frequency, regulate diet, and effectively control postprandial blood glucose and insulin levels, demonstrating that YR is a functional food. These effects may be closely related to the active ingredients in YR, including inulin-type fructans and phenolic compounds. However, the quality of the included studies was limited, and future high-quality clinical trials will be necessary to confirm the effectiveness and potential mechanism of YR.

## Data Availability

The original contributions presented in the study are included in the article/Supplementary material, further inquiries can be directed to the corresponding authors.
