# Supplementary material for: Yacon root is a functional food beneficial for human health: a meta-analysis of clinical trials
Source: Front Nutr. 2025 Dec 11;12:1739768. doi: 10.3389/fnut.2025.1739768 (PMC12738341; doi:10.3389/fnut.2025.1739768)
Supplement: Supplementary file 1 [file Image_1.pdf]

A

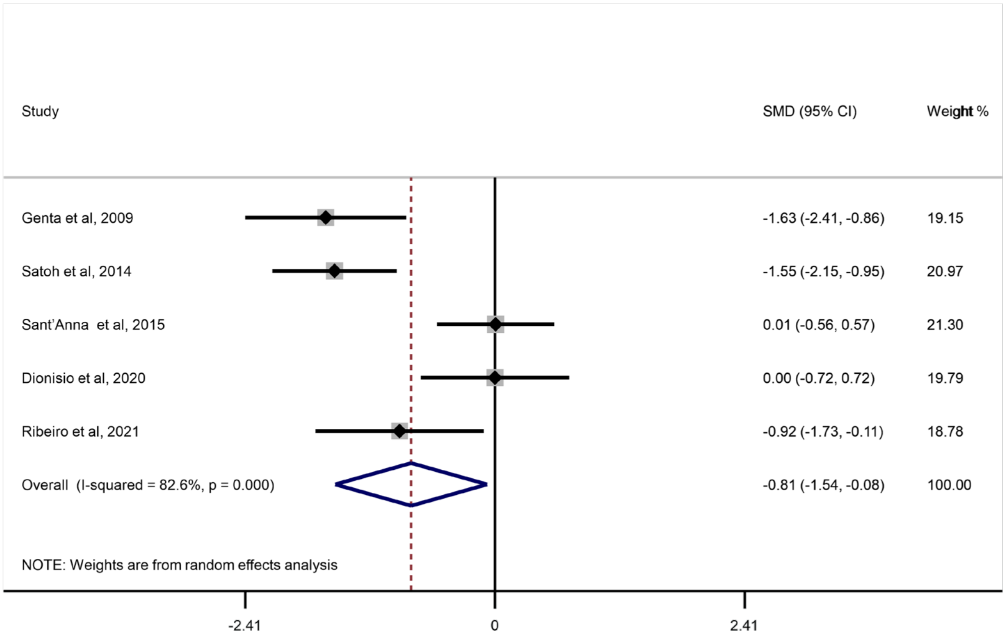

B

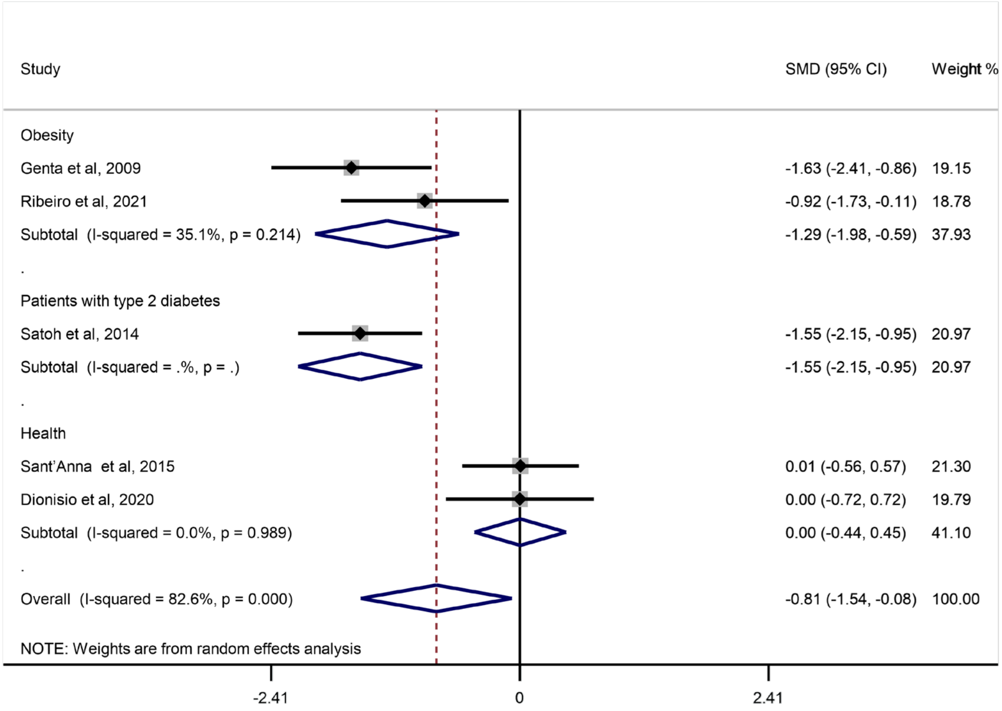

Figure S1 A. Forest plot illustrating the effect of YR on BMI; B. Subgroup analysis by subject type.

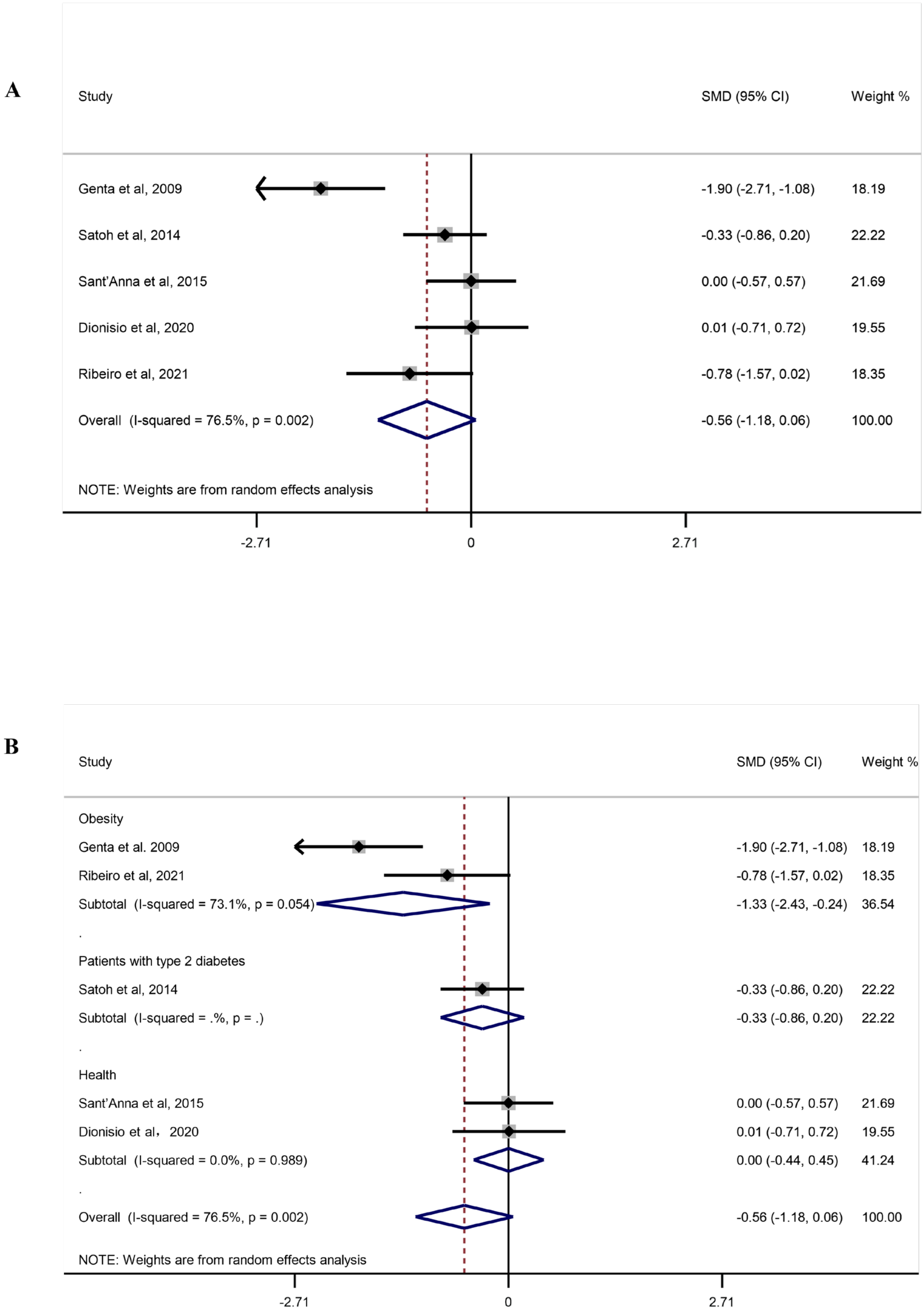

Figure S2 A. Forest plot illustrating the effect of YR on body weight; B. Subgroup analysis by subject type.

A

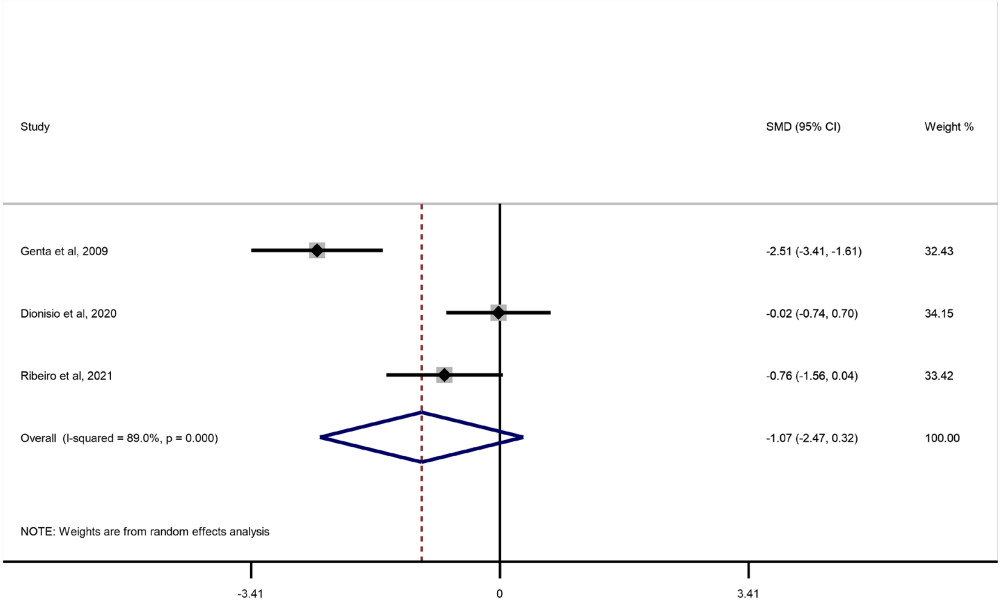

B

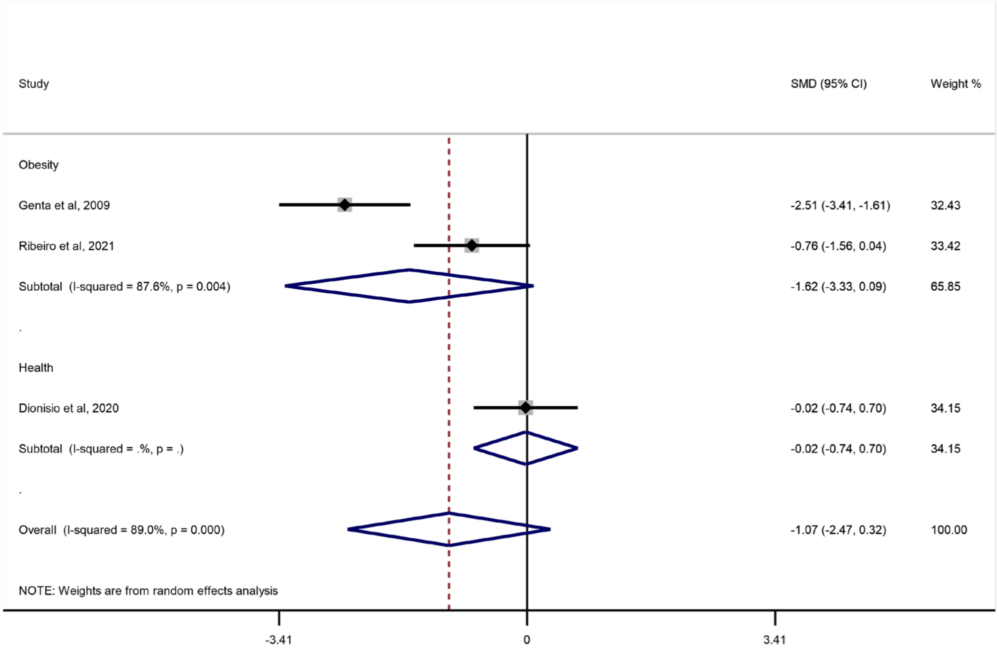

Figure S3 A. Forest plot illustrating the effect of YR on waist circumference; B. Subgroup analysis by subject type.

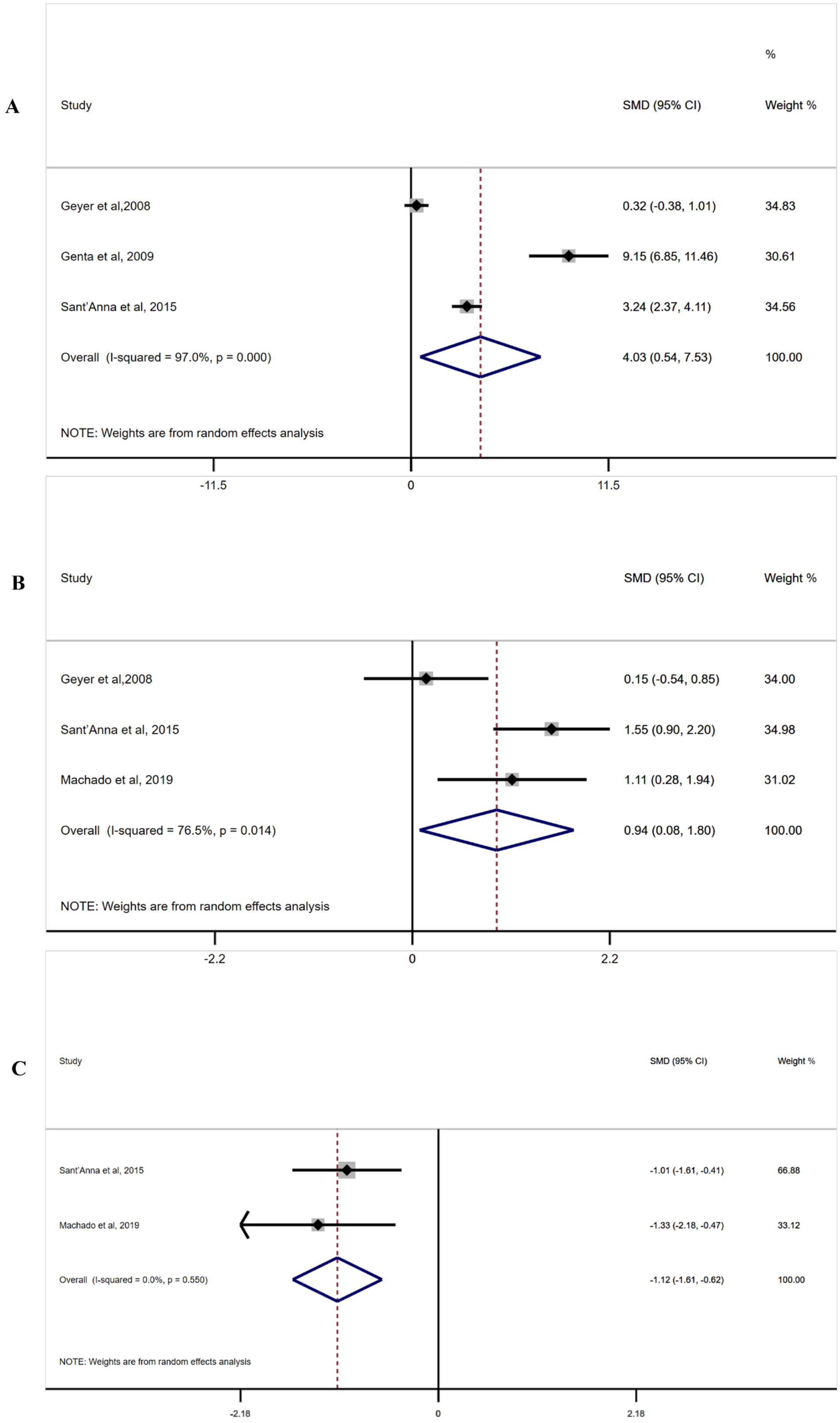

Figure S4 Forest plots showing the effect of YR on stool frequency (A), stool consistency (B), and fecal pH (C).

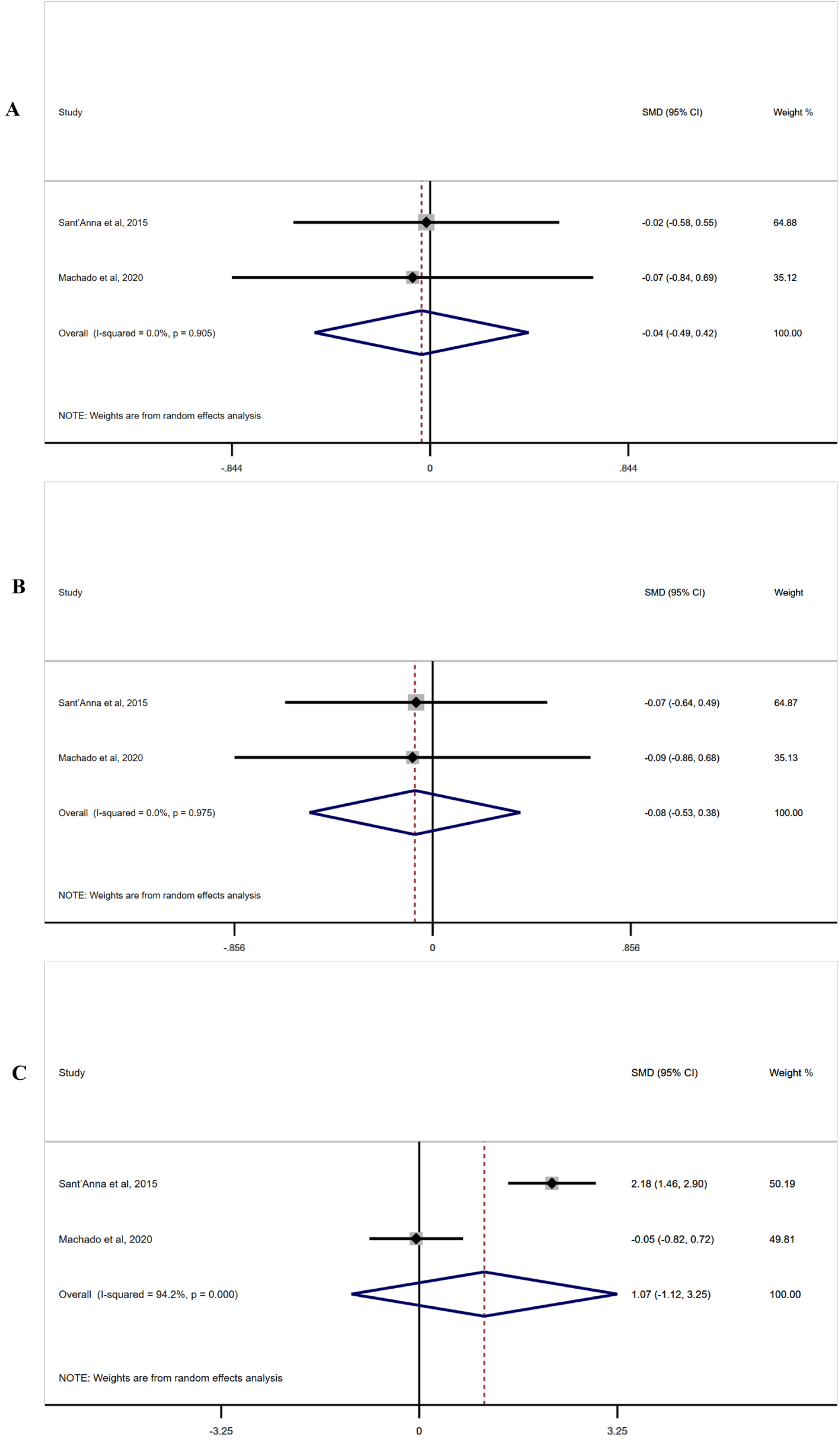

Figure S5 Forest plot illustrating the effect of YR on short-chain fatty acids: acetate (A), propionate (B), and butyrate (C).

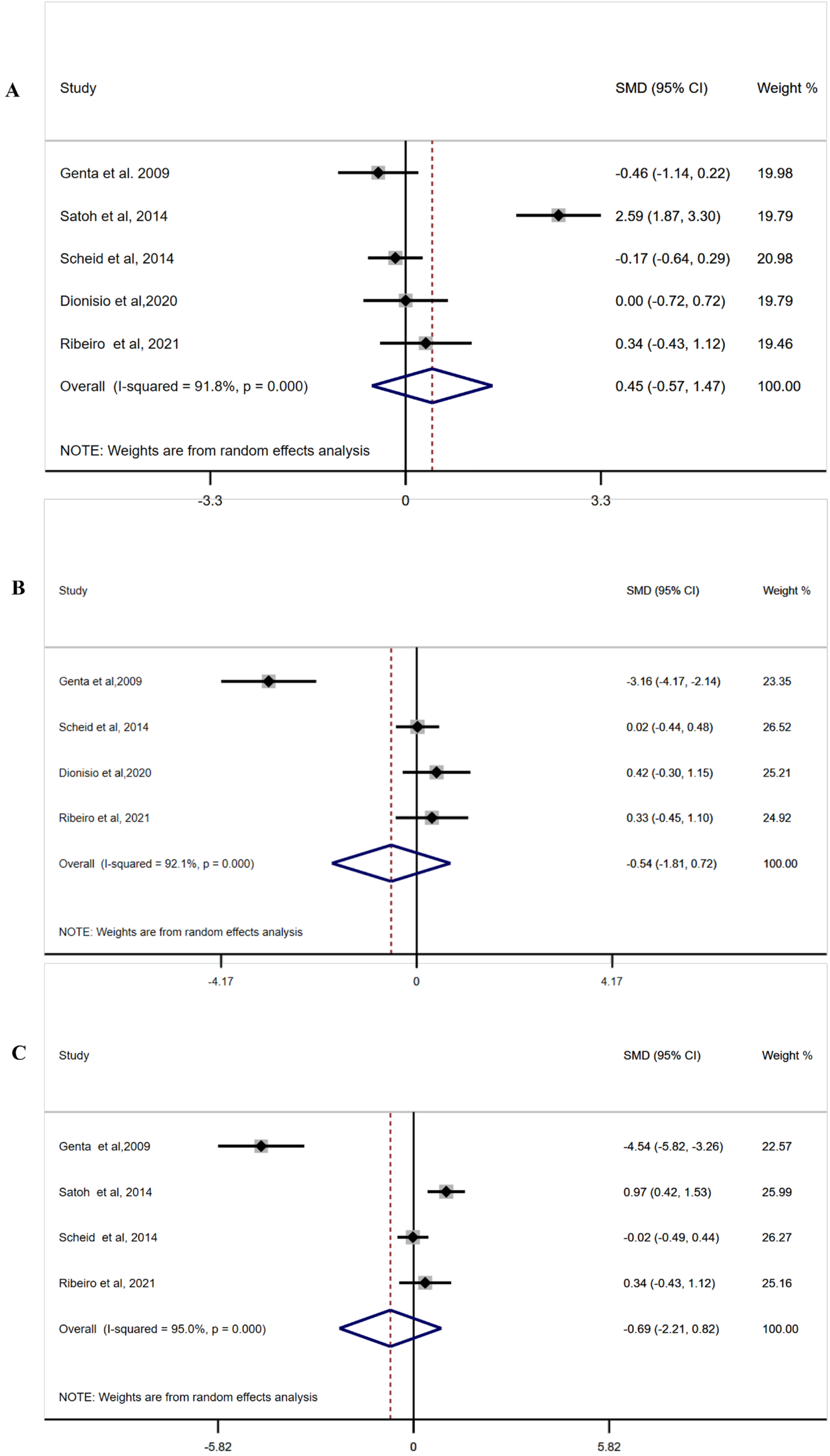

Figure S6 Forest plot illustrating the effect of YR on blood glucose (A), blood insulin (B), and HOMA-IR(C).

A

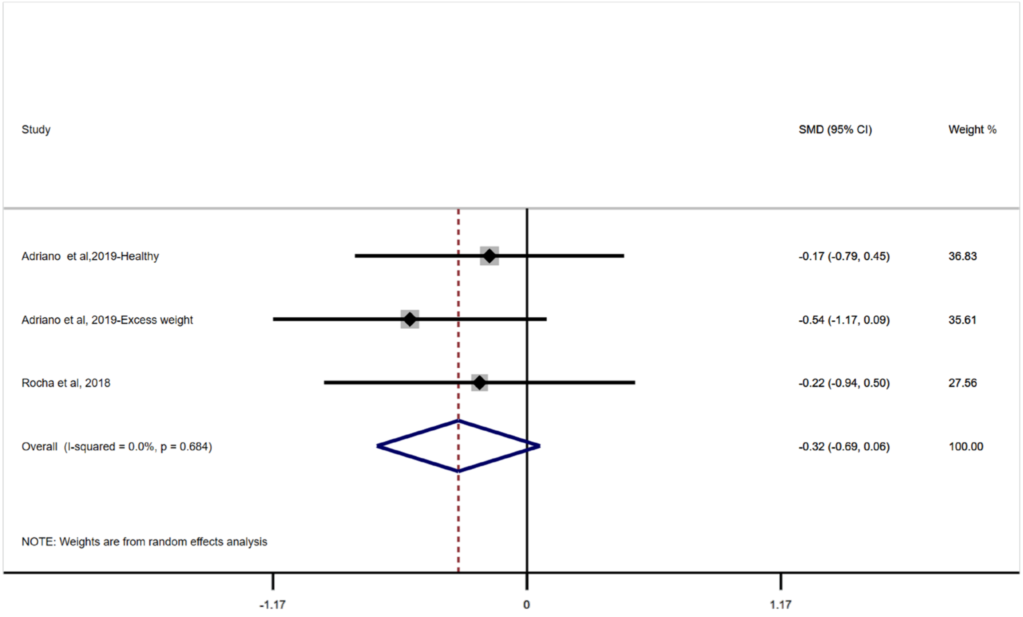

B

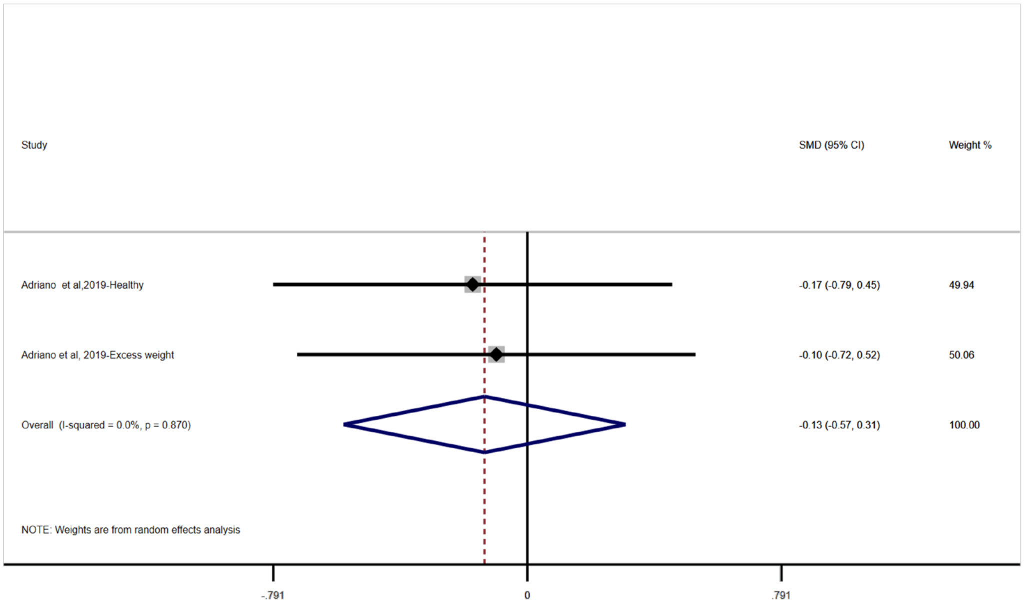

Figure S7 Forest plot illustrating the effect of YR on postprandial blood glucose (A) and postprandial triglycerides(B).

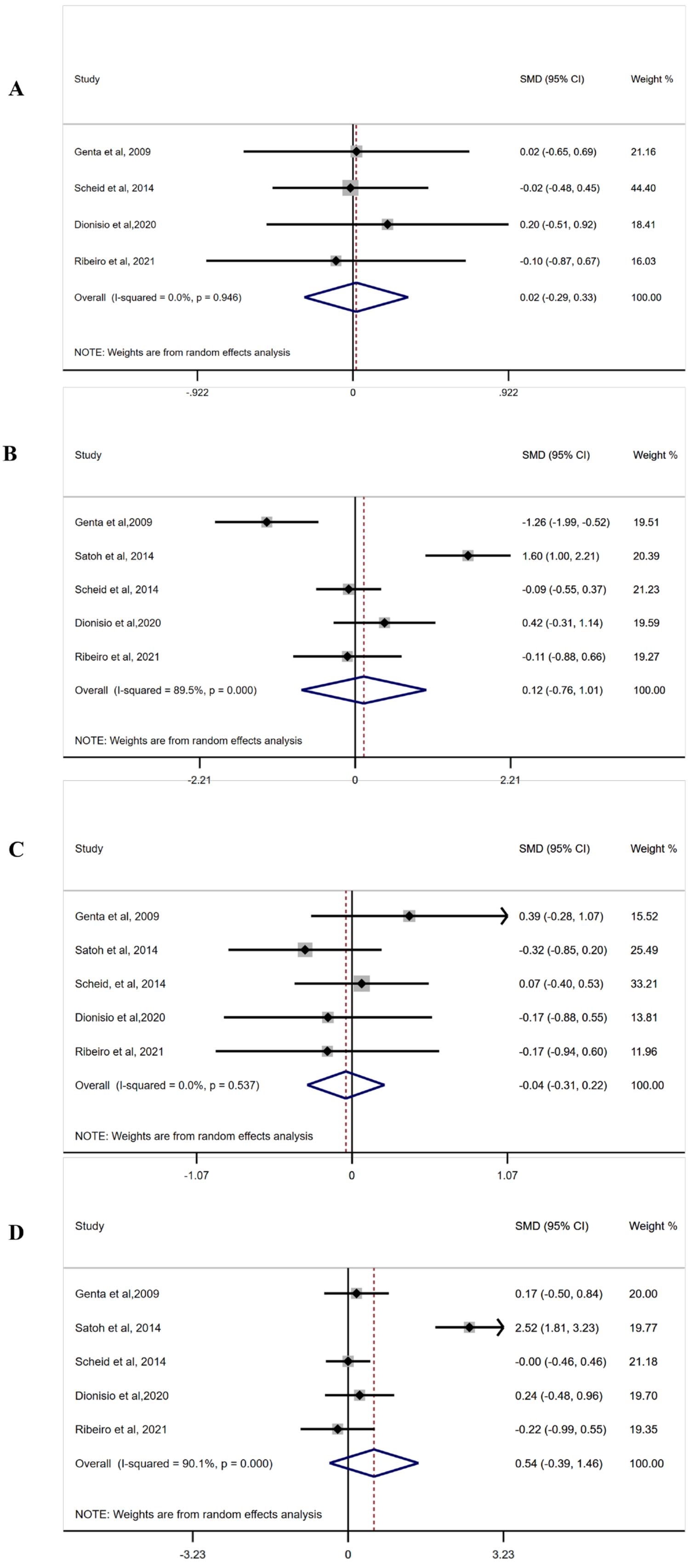

Figure S8 Forest plot illustrating the effect of YR on total cholesterol (A), LDL-c (B), HDL-c (C), and triglycerides (D).

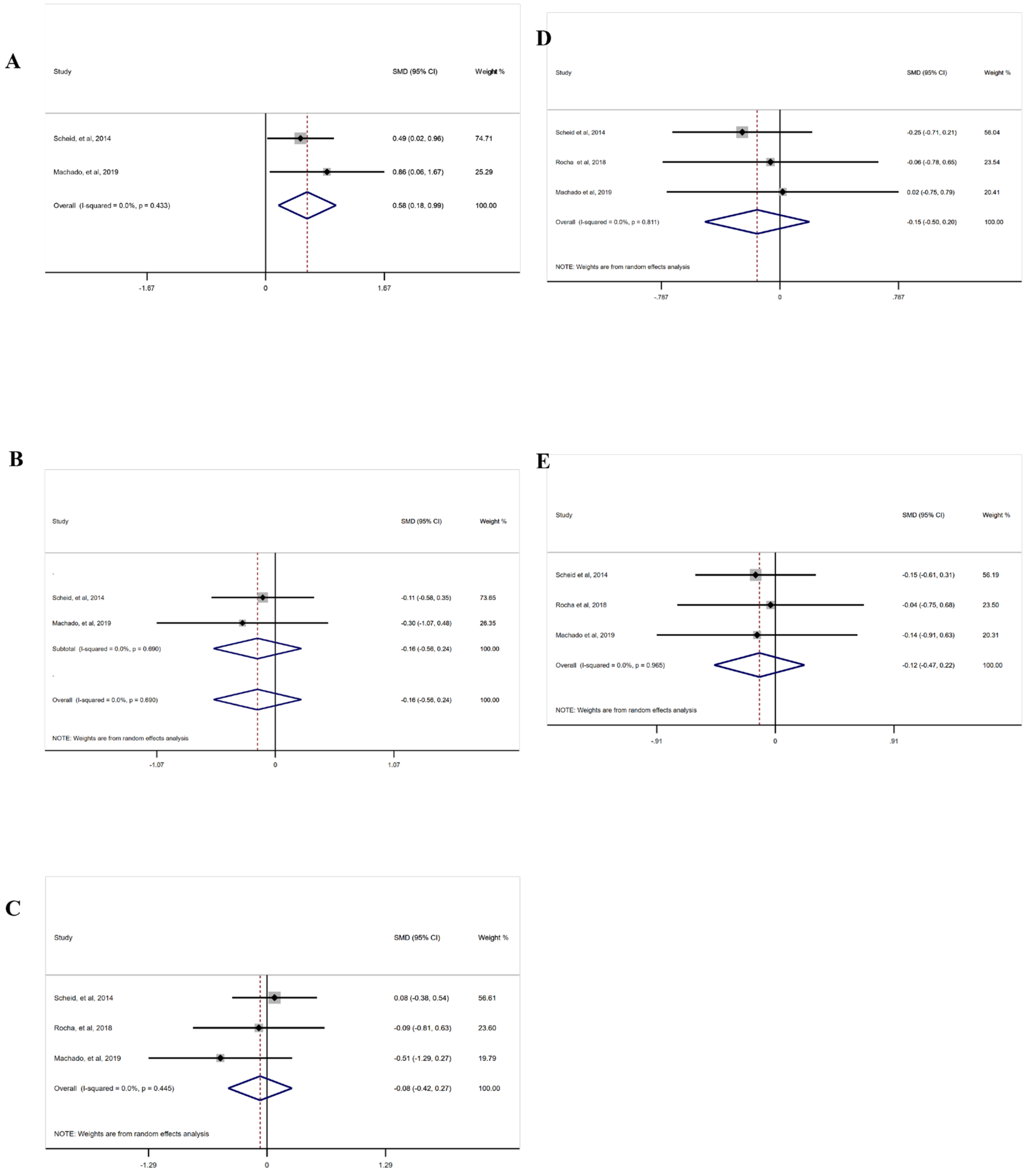

Figure S9. Forest plot illustrating the effect of YR on nutrient intake: fiber (A), energy (B), carbohydrates (C), fat (D), and protein (E).
